# Thyroid storm with concurrent autoimmune hepatitis: a case report on diagnostic challenges and the role of therapeutic plasma exchange

**DOI:** 10.1530/EDM-25-0042

**Published:** 2025-08-18

**Authors:** Mohd Hazriq Awang, Nur ‘Aini Eddy Warman, Fatimah Zaherah Mohd Shah, Aimi Fadilah Mohamad, Nur Aisyah Zainordin, Rohana Abdul Ghani, Effat Omar

**Affiliations:** ^1^ Faculty of Medicine, Universiti Teknologi MARA (UiTM), Selangor, Malaysia; ^2^ Institute of Pathology, Laboratory and Forensic Medicine, Universiti Teknologi MARA (UiTM), Selangor, Malaysia

**Keywords:** thyroid storm, autoimmune hepatitis, enchephalopathy, therapeutic plasma exchange

## Abstract

**Summary:**

Thyroid storm represents a severe expression of thyrotoxicosis and is commonly associated by multiple organ dysfunction and liver abnormality. Thyrotoxicosis with concurrent autoimmune hepatitis is rare but could confound liver dysfunction, hence complicating diagnosis and subsequent management. We present the case of a 37-year-old woman with thyroid storm complicated by multiple organ failure and resistant hyperthyroidism. Despite initial medical therapy, her condition deteriorated with rising bilirubin and worsening right heart failure. Therapeutic plasma exchange (TPE) was initiated, leading to transient clinical and biochemical improvements. However, subsequent neurological decline and hepatic decompensation revealed an underlying autoimmune hepatitis, confirmed via biopsy at later stage. Intensive multimodal management, including further TPE sessions, ultimately stabilized her condition, allowing a safe and successful thyroidectomy. This report underscores the diagnostic challenge posed by overlapping autoimmune pathologies, the need for vigilant monitoring of thyroid and hepatic parameters, and the pivotal role of TPE as a rescue therapy.

**Learning points:**

- Dual autoimmune pathologies, such as autoimmune hepatitis and Graves’ disease, can overlap and complicate diagnosis.
- Persistent or worsening liver dysfunction despite improvement in other thyroid parameter may signal underlying liver pathology.
- Importance of monitoring for rebound thyrotoxicosis and liver dysfunction in thyroid storm.
- Therapeutic plasma exchange (TPE) as a critical rescue therapy in thyroid storm and metabolic or hepatic encephalopathy.

## Background

The association between hyperthyroidism and elevated liver enzymes has been well documented (1, 2, 3, 4). Hepatic dysfunction in hyperthyroidism is often multifactorial, involving factors such as the free triiodothyronine (fT3) hormone inducing mitochondria-mediated hepatocyte apoptosis, hepatic ischaemia due to peripheral vasodilation, right-sided congestive cardiac failure leading to hepatic venous congestion and iatrogenic (2). The concurrent presence of other autoimmune diseases affecting the liver, such as autoimmune hepatitis in Graves’ disease, has also been reported, but much rare, particularly with proven histopathological findings (4, 5, 6, 7).

To the best of our knowledge, this is the first reported case of severe thyroid storm with a concurrent autoimmune hepatitis (AIH) and severe metabolic encephalopathy. In addition, this case demonstrated a significant role of therapeutic plasma exchange (TPE) in the management of this complex, intertwined and critical condition.

## Case presentation

A 37-year-old Malay woman was referred to our institution for further management of severe thyrotoxicosis. She was diagnosed with Graves’ disease 5 days prior, following a complaint of worsening shortness of breath, jaundice, abdominal swelling, and leg oedema over a period of 3 weeks. In addition, she had been experiencing intermittent palpitations, diarrhoea, and significant weight loss for the past 3 months. She had no known medical illnesses and was not taking any medications or supplements before presentation.

On admission, her blood pressure was 160/84 mmHg and she was in atrial fibrillation with a heart rate of 140 beats per minute, and temperature of 37.5°C. She appeared cachexic with weight 42 kg and body mass index 17.7 kg/m^2^. There was prominent scleral icterus, ascites and significant pedal oedema extending up to the thighs. There was evidence of dysthyroid eye disease, and a moderately sized goitre with audible bruit upon auscultation. Her cognitive function was normal and there were no focal signs of infections. The Burch–Wartofsky score was elevated at 65 (heart rate: 25 points, severe jaundice: 20 points, atrial fibrillation: 10 points, congestive heart failure: 10 points), consistent with a diagnosis of thyroid storm.

## Investigation

Laboratory investigations revealed elevated thyroxine (fT4) levels at 52.2 pmol/L (normal range: 7.8–14.4), with suppressed thyroid-stimulating hormone (TSH). The bilirubin level was significantly raised at 267 μmol/L (normal range: 0–21) with marginal increase of other liver enzyme. The coagulation profile showed severe derangement with an INR of 3.5 and a prothrombin time of 45.8 s (reference range: 11–15). Inflammatory markers were within normal limits. The TSH receptor antibody (TRab) was elevated at 3.10 IU/L (normal value: <1.75).

Thyroid ultrasound revealed an enlarged thyroid gland with heterogeneous echotexture and increased colour signal throughout the gland. Echocardiography demonstrated right-sided heart failure with severe tricuspid regurgitation, pulmonary artery systolic pressure (PASP) 50 mmHg and the ejection fraction was within the normal range of 50–60%. Ultrasound of abdomen was unremarkable and revealed no intrahepatic or common bile duct dilatation.

## Treatment

She had been initiated on an aggressive medical regimen, including carbimazole 30 mg twice daily, intravenous hydrocortisone 100 mg three times daily, cholestyramine 4 g three times daily, Lugol’s iodine 50 mg three times daily, lithium carbonate 300 mg twice daily, and bisoprolol 7.5 mg once daily. She had also received intravenous vitamin K, which partially corrected the abnormal coagulation profile.

However, a week later, the fT4 remain markedly elevated and bilirubin levels continued to rise. In view of the persistent hyperthyroidism, and worsening liver enzymes, she was planned for an urgent thyroidectomy, to be preceded by TPE to optimize her clinical status.

Three sessions of TPE were administered over 4 days, with each session lasting 2 h using fresh frozen plasma (FFP) as fluid replacement with the formula as given below:

Plasma volume=weight×0.065×(1-Hematocrit)=50 kg×0.065×(1-0.36)=2.08 FFP volume=2.08×1.5 plasma volume=3.1 L FFP

**Figure 1:**
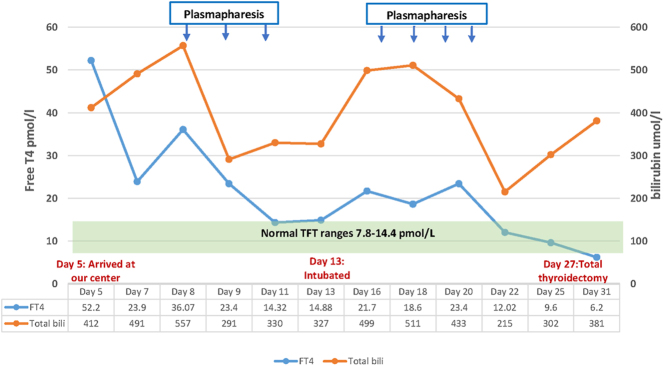
Patient’s bilirubin and fT4 course during treatment.

The intervention had effectively lowered her fT4 to normal range and serum bilirubin decreased by half to 248 μmol/L (Fig. 1; Table 1). Repeated echocardiogram showed normalization of right heart contractility and pressure, along with the resolution of the tricuspid regurgitation. This improvement suggests a completion of TPE treatment and she was referred for thyroidectomy for subsequent management.

**Table 1:** Thyroid hormone and liver enzyme before and after TPE.

Biochemical	Day 8 (before first TPE)	Day 11 (before 3rd TPE)	Day 13 coma	Day 16 (before 4th TPE)	Day 22 (after 7th TPE)	Reference range
FT4, pmol/L	36.07	14.32	14.88	21.7	12.2	7.86–14.41
TSH, mIU/L	<0.01	<0.01	<0.01	<0.01	0.03	0.38–5.33
Bilirubin, μmol/L	557	248	327	499	215	0–21
INR (based on ISI)	1.6	1.2	1.2	1.4	1.1	<1.1
PT, seconds	16.6	13.7	13.7	15	12.7	11–13.5
ALT, U/L	63	45	65	69	38	<50
AST, IU/L	43	-	78	40	61	<30
ALP, U/L	178	122	158	100	90	35–104

## Outcome and follow-up

Unfortunately, 3 days later, she reported insomnia, persistent headache, and drowsiness. This was followed by an acute episode of loss of consciousness and she was promptly intubated and ventilated.

Electroencephalography (EEG) demonstrated widespread generalized slowing at 3 Hz, with frequent, symmetrical, synchronous paroxysmal lateralized epileptiform discharges (PLEDs), suggestive of non-convulsive status epilepticus. Intravenous levetiracetam and phenobarbital were administered. Brain MRI demonstrated nonspecific sulcal hyperintensity with mild cortical swelling in the bilateral parieto-occipital regions, prompting a broad differential diagnosis, including encephalitis and metabolic insult. Autoimmune encephalitis was considered and empirical treatment with intravenous methylprednisolone (500 mg/day for 5 days) was initiated. However, this diagnosis was considered unlikely after cerebrospinal fluid analysis and relevant blood tests returned unremarkable findings, coupled with the absence of clinical response to pulse steroid therapy.

Further blood tests showed a significant rise in ammonia levels at 218 μmol/L (normal range: 11–32 μmol/L) and a positive anti-smooth muscle antibody. Other relevant liver investigations, including hepatobiliary ultrasound, viral hepatitis screening, anti-mitochondrial antibodies, and anti-nuclear antibodies (ANA), were normal. Dexamethasone was initiated after completion of methylprednisolone to cover for autoimmune hepatitis. She also received prophylactic therapy for hepatic encephalopathy including lactulose and thiamine.

Her condition deteriorated further with worsening coagulopathy, lower gastrointestinal bleeding, and hospital acquired pneumonia and arrhythmia (atrioventricular nodal re-entrant tachycardia and atrial fibrillation). In addition, worsening blood parameters, particularly bilirubin and rising fT4 levels, prompted the decision for the continuation of TPE. Four additional TPE sessions were administered (Fig. 1). Following the second course of TPE, her GCS improved from 3/15 to 14/15. By the completion of 4th TPE, her fT4 had normalized again, and bilirubin had reduced more than half to 215 μmol/L. In addition, the ammonia level dropped from 218 to 63 μmol/L (normal range: 11–32) (Table 1).

The improving condition following TPE provided the opportunity to proceed with definitive surgical intervention. Thyroidectomy was eventually performed on day 27 of hospital admission. The procedure was uneventful. Her prolonged hospital stay was complicated by critical illness polyneuropathy, which required intensive rehabilitation. She was discharged home a month later in a semi-dependent state. She was able to return to basic normal activities 6 months after her discharge.

**Figure 2:**
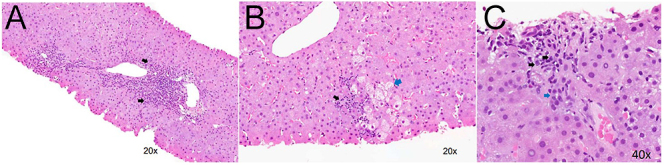
Photomicrograph of the liver biopsy from the patient. (A) Area of severe lymphocyte and plasma cell infiltration of the portal tract, spilling past the limiting hepatocyte plate into the hepatic lobule (interface hepatitis) (black arrows). (B) Spotty inflammatory cell infiltration (black arrow) is seen in the lobule. Some hepatocytes are ballooned (blue arrow), indicating cell damage. (C) High power view demonstrating plasma cells (black arrow). A bile duct is seen on the side (blue arrow), indicating that this is a portal tract.

Her liver functions had recovered gradually and serum bilirubin approaching normal range while continuing low dose steroid. However, persistent and unexplained elevated transaminases and alkaline phosphatase necessitated a liver biopsy. Histopathological investigation revealed mild chronic hepatitis and periportal fibrosis with Batts–Ludwig system grading and staging score of 2, supportive of the diagnosis of autoimmune hepatitis (Fig. 2).

## Discussion

This report illustrated a rare presentation of thyroid storm with concurrent autoimmune hepatitis and severe hepatic encephalopathy. It highlighted the diagnostic challenge in recognizing these coexisting autoimmune diseases that have overlapping clinical features, in particular liver manifestation, and the subsequent complex and difficult ensuing treatment.

The pattern of hepatic dysfunction in thyrotoxicosis varies widely, ranging from mild transaminitis to significant cholestatic dysfunction, but incidence of acute liver failure remains uncommon (1, 3, 4, 5). One notable abnormality of concern is elevated bilirubin, with an incidence reported between 17.6% and 21.3% in thyroid storm cases (5) and associated with poorer prognosis when total bilirubin level exceeds 51 μmol/L (3).

Association between Graves’ disease and autoimmune hepatitis (AIH) has been rarely described in the literature (4, 5, 6, 7). The estimated prevalence of Graves’ disease in autoimmune hepatitis is around 6%, double compared to the general population (4). Both diseases are known to be immune mediated, but the exact mechanism of association remains unclear.

In the most recently published cases of concurrent Graves’ disease and AIH, all patients were female, with ages ranging from 12 to 58 years (5, 6, 7). However, the concurrent identification of both Graves’ disease and AIH at initial presentation was only established in four out of 13 cases (5, 6). Patients in the case reports exhibited symptoms of hyperthyroidism, accompanied by significantly elevated transaminase levels, exceeding 20–50 times upper limit of normal, which prompted earlier investigation and subsequent early diagnosis of AIH.

Graves’ disease preceded the diagnosis of AIH in five other cases, ranging from a few months up to 12 years apart (5, 6). Four of these patients were treated with anti-thyroid drug (ATD) without any complication, in which two of them had existing elevated liver enzymes at the initial diagnosis of Graves’ disease. In this group, the persistent elevation of liver transaminases led to further investigations and the subsequent diagnosis of AIH. AIH was identified before the diagnosis of Graves’ disease in the remaining four cases, the duration ranging from 9 months to 16 years earlier (5, 6, 7).

To the best of our knowledge, our patient is the first reported case of early presentation of AIH during thyroid storm. Interestingly, her liver transaminase levels were only mildly elevated (2–3 times upper limit of normal) but with severe hyperbilirubinaemia. This abnormality persists despite improvement in right heart failure and other thyrotoxicosis parameter and later required prolonged steroid treatment after thyroidectomy.

TPE has been exceedingly acknowledged as an alternative therapy for patients who either do not respond to aggressive medical therapy including ATD therapy or have contraindications to these medications (1, 3). While there are currently no prospective studies on the use of TPE in thyroid storm, increasing clinical evidence, derived from growing number of case series worldwide, support its utilization as a treatment option for complicated thyroid storm patients. The latest Japan Thyroid Association (JTA) guideline recommends TPE for thyroid storm cases that fail to show improvement within 24–48 h of initial treatment. TPE is also strongly indicated for patients with significant liver involvement, particularly elevated bilirubin levels above 51 μmol/L (3). Thus, the indication for TPE in our patient was consistent with both recommendations on multiple perspectives.

Our patient exhibited marked clinical improvement following initial three sessions of TPE, with normalization of fT4 levels and improvement in liver function parameters. However, her subsequent clinical deterioration, most notably the development of significant neurological sequelae, presented a diagnostic and therapeutic challenge.

The rapid rebound in liver enzymes, particularly bilirubin, accompanied by elevated serum ammonia levels, raised suspicion of a persistent hepatic insult beyond the initial thyrotoxic state. Although relapse of thyroid storm remained a differential consideration, the progression of symptoms despite normalization of fT4 and stabilization of other thyroid parameters suggested an alternative or concurrent hepatic pathology. Subsequent investigations supported a working diagnosis of additional liver injury, possibly attributable to autoimmune hepatitis.

Fortunately, additional courses of TPE did ameliorate her condition without long-term consequences. It remains uncertain whether a more extended course of TPE from the outset beyond the initial three sessions might have prevented this sequence of events or altered the patient’s clinical trajectory. Currently, evidence guiding the optimal frequency and duration of TPE in thyroid storm is limited, and its application remains largely based on clinical judgement and serial fT4 measurements (1, 3).

The efficacy of TPE lies in its capability to eliminate excess catecholamines, cytokines, thyroid hormone, and TRAb (8, 9). In a particular case series, TPE demonstrated reductions in plasma fT4 by 31.6–66% and TRAb levels by more than 50% after the initial TPE cycle (9). In other reports, when used as rescue therapy before urgent thyroidectomy, TPE showed an average reduction of fT3 by 65–83%, fT4 by 22–66%, and TRAb by 55–96% after a median of three TPE sessions (8). Similar observation showed in our patient with 61% decrease in fT4 levels after the initial three sessions of TPE. This biochemical improvement was also swiftly accompanied by clinical recovery, including the resolution of her cardiac function and improvement in her liver enzyme levels.

The utilization of TPE has also been widely documented in acute liver failure. Its benefit has been proven in the randomized-control trial in acute liver failure, showing increasing liver transplant-free survival, and, in addition to reducing ammonia levels, improves hepatic encephalopathy grades (10). Our patient demonstrated consistent results in terms of reductions in her bilirubin and ammonia levels, and consequently leading to significant improvement in encephalopathy.

## Conclusion

The association between Graves’ disease and AIH is uncommon, although both conditions present with apparent elevated liver enzymes. This report underscores the importance to consider the diagnosis of AIH in patients with Graves’ Disease, complicated by elevated liver enzymes, which could compromise the optimum use of ATD. AIH should be considered in patients with persistently raised liver enzymes despite improvements in thyroid hormone levels, or with severe elevation in transaminase levels especially exceeding 20 times the upper limit of normal. This report also demonstrated that TPE is an effective and safe treatment in severe thyroid storm, particularly complicated by acute liver failure.

## Declaration of interest

The authors declare that there is no conflict of interest that could be perceived as prejudicing the impartiality of the work reported.

## Funding

This work did not receive any specific grant from any funding agency in the public, commercial, or not-for-profit sector.

## Patient consent

Written informed consent was obtained from the patient for publication of this case report.

## Author contribution statement

MHA is the main author and physician involved with the patient’s care. NAEW, FZMS, RAG and AFM were the endocrine physicians involved with the patient’s care. NAZ was the medical physician involved with patient’s care. EO was the pathologist involved with patient’s histopathology analysis.
